# Reducing Organic Load From Industrial Residual Process Brine With a Novel Halophilic Mixed Culture: Scale-Up and Long-Term Piloting of an Integrated Bioprocess

**DOI:** 10.3389/fbioe.2022.896576

**Published:** 2022-04-19

**Authors:** Thomas Mainka, Christoph Herwig, Stefan Pflügl

**Affiliations:** ^1^ Institute for Chemical, Environmental and Bioscience Engineering, TU Wien, Vienna, Austria; ^2^ Competence Center CHASE GmbH, Linz, Austria

**Keywords:** bioprocess scale-up, halophilic microbial community, industrial residual process brine treatment, aromatic degradation, long-term bioprocessing

## Abstract

Integrating bioprocess solutions for treatment and subsequent reuse of saline residual process brine into industrial processes could increase the sustainability of production chains. However, such bioprocesses require large-scales and a robust operation over a prolonged period. Consequently, the aim of this study was to analyze scale-up equivalence as well as continuous and stable process performance of a previously established lab scale process for the degradation of organic contaminants (formate and aromatic compounds) in an industrial context. To that end, a pilot-scale bubble column bioreactor system equipped with a membrane-based cell retention system for process intensification was integrated at an industrial production site. The process was successfully scaled-up and continuously operated for more than 210 days. Overall, the process proved to be robust towards changing compositions of the residual process brine stream and degradation rates for organic contaminants were close to 100%. Interestingly, due to the unsterile process conditions, the original *Haloferax mediterranei* culture was replaced by a novel halophilic bacterial community consisting of three bacterial genera. To further improve process economics and productivity, an optimization of the co-substrate feeding strategy for glycerol is required, as results indicated a potential correlation between glycerol feeding and formate degradation rates. To that end, decoupling of the glycerol feeding from the residual process brine feed is a potential way to increase process control options and allow for easy adaptation of the process to changing residual process brine compositions. Ultimately, the process described here could be a promising alternative for chemical or physical methods of treating residual process brine and once more underlines the potential to exploit natural microbial diversity for industrial purposes.

## Graphical Abstract

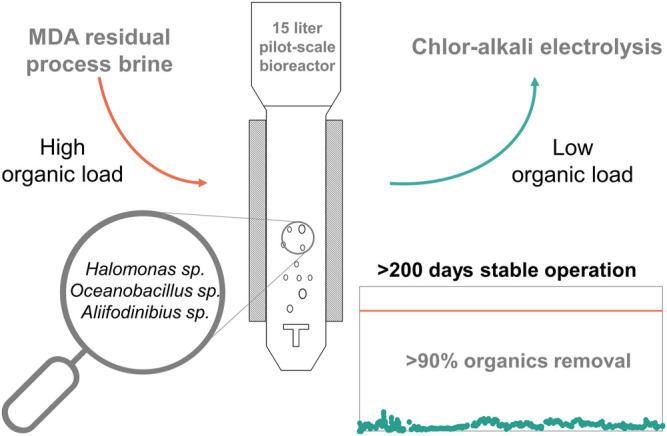

## 1 Introduction

In various industrial sectors, salt- and non-salt-containing residual process brine (RPB) are generated. Before RPB can be released to the environment, treatment is both necessary and challenging (Lefebvre and Moletta, 2006; Prasse et al., 2015; Crini and Lichtfouse, 2018). However, NaCl containing RPB is an excellent source for a more sustainable production of chlorine and sodium hydroxide (Du et al., 2018). Therefore, the use of RPB to produce basic chemicals offers a huge potential in making industrial production chains more sustainable and cost effective (Särkkä et al., 2015; Muddemann et al., 2018). Nevertheless, organic impurities in NaCl-containing RPB have a negative effect on membranes used in chlorine-alkali processes, since they can cause precipitates and foaming, resulting in potential voltage increases (Keating and Behling, 1990; Silva et al., 2009). Therefore, brine treatment is necessary to avoid an inefficient membrane process and even a damage to the membrane. Physical and electrochemical approaches for the reduction of organic impurities in chlorine-alkali brines have already been reported (Heuser et al., 2005; Muddemann et al., 2018; Bulan et al., 2019).

A promising alternative for the degradation of organic contaminants in RPB can be the treatment with halophilic microorganisms able to degrade the substances contaminating streams such as RPB (Bertrand et al., 1990; Alva and Peyton, 2003; Le Borgne et al., 2008; Tapilatu et al., 2010; Erdoğmuş et al., 2013; Castillo-Carvajal et al., 2014; Corti Monzon et al., 2018; Jamal and Pugazhendi, 2018; Aron et al., 2021; Mainka et al., 2021). However, the integration of biological unit operations into chemical production chains is often limited by the complexity of bioprocesses. Special nutrient supplementation, the risk of contamination and the widespread discontinuous process mode (mostly batch or fed-batch processes in biotechnology) are major concerns of the chemical industry. Processes developed with halophilic microorganisms have the potential to overcome several of the above-mentioned limitations. Due to high salt conditions, the risk of contamination is low (Schiraldi and De Rosa, 2002; Bonete and Martínez-Espinosa, 2011; Deive et al., 2012). Moreover, it has already been shown that nutrient supplementation can be kept to a minimum (use of minimal medium) and the use of easily accessible and renewably produced carbon sources such as glycerol is possible (Berrios and Skelton, 2008; Pflügl et al., 2014; Russmayer et al., 2019). In contrast to chemical processes, biological processes operated in continuous mode are perceived to bear an increased risk of instability, either genetically or through a decrease of specific productivity (Deive et al., 2012). Furthermore, continuous bioprocessing demands a more complex process setup. Nevertheless, it has already been shown for halophilic microorganisms, that continuous bioprocessing in lab-scale is successful and applicable for RPB treatment processes (Fallet et al., 2010; Mahler et al., 2018; Mainka et al., 2019).

However, academic studies frequently are time- and space-restricted. As a result, no studies are available which investigated long-term culturing effects on process performance of pilot-scale biological RPB treatment processes using halophilic microorganisms. The aim of this study was to establish a pilot-scale bubble-column fermentation system for the continuous biological treatment of RPB directly implemented at an industrial production site. The high-salt RPB contains the organic contaminants formate, aniline, phenol and 4,4’-methlyendianiline (MDA), which are substrates and products of the industrial MDA production. To provide RPB suitable for subsequent sodium chloride production, halophilic microorganisms are interesting biocatalysts. Degradation of the organic impurities by halophiles has already been demonstrated for several strains, including *Haloferax mediterranei*, *Halomonas* strain MA-C, *Haloarcula* sp. A235, *Oceanimonas* sp., or *Halomonas organivorans* (García et al., 2004; Li et al., 2010; de Lourdes Moreno et al., 2011; Acikgoz and Ozcan, 2016; Tan et al., 2017; Mahler et al., 2018; Heckroth et al., 2019; Mainka et al., 2019; Mainka et al., 2021). Consequently, this study compares process performance of a lab-scale and pilot-scale halophilic bioprocessing system to remove organic compounds from RPB. In order to extend the previously gained process knowledge from a lab-scale bioprocess using the extremely halophilic archaeon *H. mediterranei* was scaled-up to a bubble column bioreactor setup (Mahler et al., 2018; Mainka et al., 2019). Moreover, for the first time, such a pilot-scale bioreactor system was set up, implemented, and integrated in an industrial MDA-production site. To measure the success of the scale-up, process variables (dilution rate D, retention rate R, and substrate feeding) and process performance (degradation efficiency) of the lab-scale and pilot-scale process with *H. mediterranei* were compared.

In addition to comparing process scales, we investigated batch-to-batch variations of organic contaminant concentrations and their influence on the bioprocess. For industrial MDA-processes it is known that the RPB underlies variations in its composition as production conditions can change. However, in lab-scale experiments only a low number of RPB batches were used, so potential effects of changing feed conditions were not investigated. Thus, an advantage of the local integration of the bioprocess setup in the chemical production plant demonstrated in this study is the direct use of RPB without storage time between different RPB batches.

## 2 Materials and Methods

### 2.1 Bioreactor Setup

**FIGURE 1:**
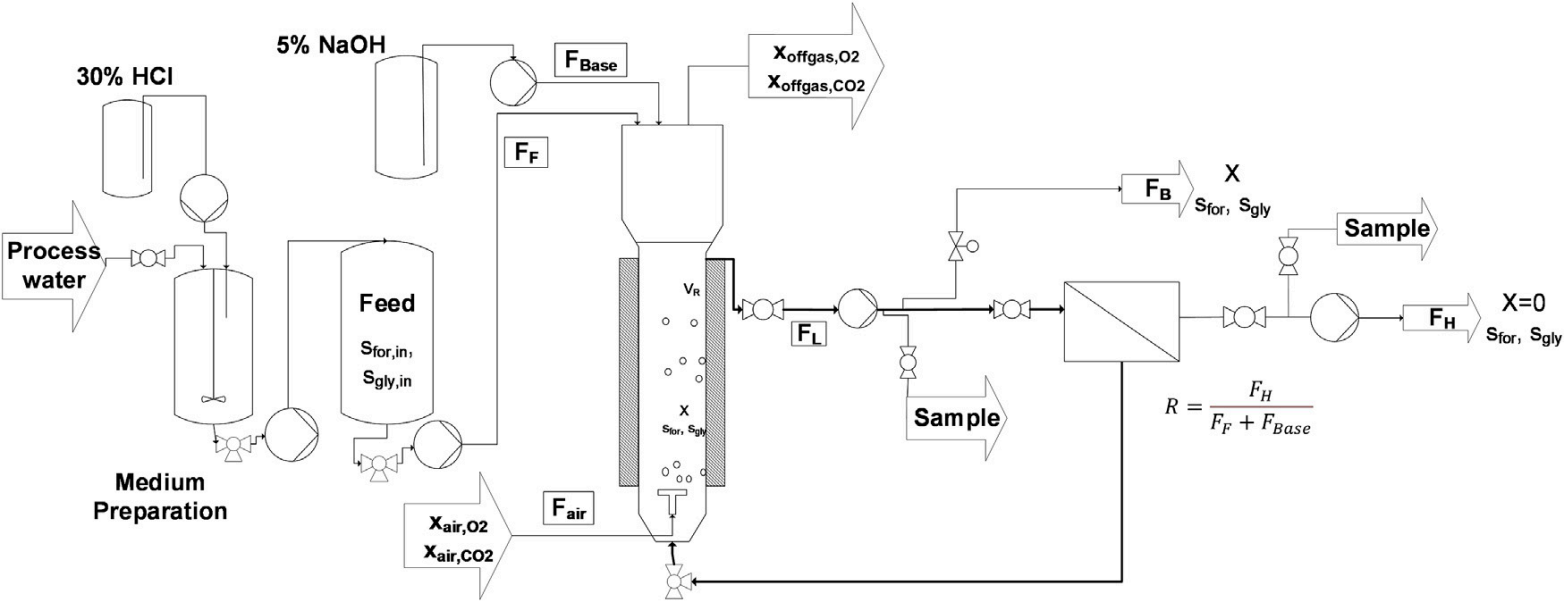
Scheme of the fermentation setup. Residual process brine from the MDA production is prepared and supplemented with the required components in a first 200 L vessel. From there the medium is pumped into the feed vessel, from where a constant feed (FF) supplies the cells with fresh substrate and media components. 5 % NaOH (FBase) is added to hold the pH on a constant level of 7.0. A pump continuously circulates the cell suspension as loop flow (FL) through the membrane module to separate cell-free harvest (FH). The ratio of outgoing harvest flow to the total iput flow is called the retention rate R and is calculated according to the following equation: $R=\;\frac{F_{H}}{F_{F}+F_{\mathrm{Base}}}$ . Bleed flow (FB) is continuously removed to eliminate cells and sustain steady state conditions. To guarantee constant reactor volume (VR) flows for Feed, Base, Harvest and Bleed have to meet the following equation: FF + FBase = FH + FB. Biomass is monitored using a turbidity probe and a soft sensor that is driven by measurements of off-gas composition.

The bioreactor setup, where the cultivations were performed, is a corrosion-resistant, custom-made bubble column reactor, with a total volume of 21 L (Diameter 13.4 cm; Height 1.1 m; Möstl Anlagenbau, Arzberg, Austria) (Mahler et al., 2018). The bioreactor consists of Hastelloy-C22 extended with a loop where all piping, valves, and screwing are made of PVDF or PTFE. The loop also contains a membrane-based cell retention unit (Hollow Fiber Cartridge, CFP-2-E-9A, 0.2 µm, 8400 cm2, GE, Westborough, United States) and a 4-piston diaphragm loop pump (Quattroflow, ALMATEC Maschinenbau, Germany) with a polypropylene pump head for circulation the cell suspension with a flow of around 100 L h^−1^. A schematic diagram of the experimental setup is shown in Figure 1.

The bleed was released with a digitally operated valve (Bürkert, Germany) into the bleed vessel and worked as a control valve to keep the reactor volume constant. The filtrate was withdrawn through the membrane, using a peristaltic pump (ISMATEC Reglo Quick, Cole-Parmer, United States). The input flows to the bioreactor system were controlled with an analog pump (Magdos, Lutz-Jesco GmbH, Wedemark, Germany) to continuously add fresh medium and a digital pump (Magdos, Lutz-Jesco GmbH, Wedemark, Germany) to add base (5% NaOH) for adjusting the pH. The flow rates for feed and harvest were calculated based on gravimetric measurements with a resolution of 10 g (Mettler Toledo, United States). Flow rates of base and bleed were determined with a resolution of 0.1 g (Kern & Sohn GmbH, Balingen-Frommern, Germany).

The inlet airflow was kept constant at 3.2 L min^-1^ (0.2 vvm) *via* a mass flow controller (Brooks Instrument, United States). Dissolved oxygen was measured using an Oxyferm probe (Visiferm DO Arc 120, Hamilton, Bonaduz, Switzerland) placed in the loop piping. To increase oxygen transfer into the liquid phase, the pressure was kept constant at 2 bar with an electronic valve (Bürkert, Germany). The pH was measured using an Easyferm pH probes (InPro3250i, Mettler Toledo, Germany), which was placed inside the loopline. The temperature probe (Onigrad TR88, Endress + Hauser, Reinach, Switzerland) was placed on the reactor vessel wall to measure the temperature inside the reactor. In the bioreactor headspace, and in the feed, retentate and filtrate sides of the membrane, sensors were placed to measure the pressure (Signet 2450, Georg Fischer, United States). The composition of off-gas was determined using a BlueVary gas analyzer system (BlueSens, Herten, Germany). For preparing the RPB medium, a 200 L stirred vessel (Schwarzer Rührtechnik, Delmenhorst, Germany) made from polypropylene was used. The final feed was then transferred to second 200 L feed vessel, from which the fermenter was continuously fed.

### 2.2 Strain and Medium

*Haloferax mediterranei* DSM 1411 was obtained from DSMZ (Deutsche Sammlung für Mikroorganismen und Zellkultur, Braunschweig, Germany). The preculture was cultivated in a 1 L lab-scale CSTR to a biomass concentration of around 8 g L^−1^ and cells were then transferred to the production site of the industrial partner. As 1 L of preculture was added to 15 L medium in the described pilot-scale bioreactor, the initial biomass concentration was 0.5 g L^−1^. To increase the level of biomass, a batch cultivation was carried out followed by a continuous cultivation to reach a biomass concentration of 7–8 g L^−1^. All cultivations were performed at a temperature of 30°C and at pH 7.0.

For the medium, RPB from an industrial partner was directly used from the RPB columns. The RPB contained 150 g L^−1^ NaCl and formate as an organic contaminant in a concentration range of 200 ± 20 mg L^−1^. After adjustment of the pH from 13 to 4 with 30 % HCl, the RPB was supplemented with mineral media components in the following concentration (g L^−1^): NH_4_Cl 1; KH_2_PO_4_ 0.15; FeCl_3_ 0.005; MgCl_2_ · 6 H_2_O 1.3; MgSO_4_ · 7 H_2_O 1.1; CaCl_2_ · 2 H_2_O 0.55; KCl 1.66; Trace elements solution 1 ml [(mg/100 ml): FeSO_4_ · 7 H_2_O 139; CuSO_4_ · 5 H_2_O 100; CoCl_2_ · 2 H_2_O 44; ZnSO_4_ · 7 H_2_O 86]; Manganese stock 1 ml [(mg/100 ml): MnCl_2_ · 4 H_2_O 18]. Glycerol was added as a substrate in concentrations of 1.5-2.5 g L^−1^. Agar plates consisted of the same media components, but agar-agar in a concentration of 15 g L^−1^ was added. All shake flask experiments were performed in 500 ml shake flaks without baffles and a liquid volume of 100 ml Samples from the shake flasks were taken under sterile conditions.

### 2.3 Calculations

#### 2.3.1 Continuous Reactor Setup

The mathematical description of the continuous fermentation system used in this study has already been published (Mahler et al., 2018). The theory of chemostat cultures extended with cell retention was also described previously (Pirt and Kurowski, 1970).

Steady-state conditions was achieved by keeping the hydraulic dilution rate D and the retention (or recycle) rate R constant. D can be calculated according to Eq. 1. The retention rate R describes the ratio of cells retained within the bioreactor and were calculated using Eq. 2. As the reactor volume VR was kept constant during the process, volumetric input and output flows were equal (see Eq. 3). The volumetric flow rates for feed (F_F_), harvest (F_H_), bleed (F_B_) and base (F_Base_) were calculated based on online balance signals.

$$D=\frac{F_{F}+F_{Base}}{V_{R}}\;\;$$ (1)

$$R=\frac{F_{H}}{F_{F}+F_{Base}}=\frac{\left(F_{F}+F_{Base}\right)-F_{B}}{F_{F}+F_{Base}}$$ (2)

$$F_{F}+F_{Base}=F_{B}+F_{H}$$ (3)

#### 2.3.2 Rate Calculations

The specific growth rate µ in shake flask experiments was calculated for the exponential growth phase using optical density measurements at a wavelength of 600 nm (OD_600_).

In the case of a steady state, the biomass concentration is constant in a cell retention setup, thus, according to Eq. 4, the specific growth is dependent on D and R. Substrate uptake rates rS can be calculated using concentration values and liquid flow rates, as described in Eq. 5.

µ=(1−R)⋅D (4)

$$r_{S}=F_{F}\cdot \left(s_{in}-s\right)$$ (5)

### 2.4 Analytical Procedures

The samples were taken on the MDA production site and meanwhile stored at -20°C. After transport to the laboratories of the TU Wien, the samples were then further analyzed.

Substrate quantification in the feed and harvest samples was done as described previously (Erian et al., 2018), using an HPLC (Vanquish UHPLC systems, Thermo-Fisher, United States) with an Aminex HPX-87H column (Bio-Rad, United States) at 60°C, an isocratic eluent of 4 mM sulfuric acid in Milli-Q water with a flow of 0.6 ml min^-1^ followed by UV detection at 210 nm and RI detection (RefracoMax520, ERC, Germany). In brief, samples were centrifuged, and the supernatant was mixed with 40 mM sulfuric acid (9:1) before 10 µl was injected in the HPLC. The samples were analyzed for residual formate and glycerol, as well as the formation of organic acids. The standards, used for quantifications, were prepared the same way as the samples and mixed with 40 mM sulfuric acid (9:1).

For the quantification of aromatic compounds in feed and harvest samples, a reversed-phase HPLC measurement (Vanquish UHPLC systems, Thermo-Fisher, United States) was carried out, using an AcclaimTM PolarAdvantage column (Thermo Scientific, United States, C16, 3 µm, 120 Å, 4.6x150 mm) at 30 °C. Aromatic compounds were detected using a UV detector at 210 nm. The flow was 1 ml min-1 with a gradient system (0–2.5 min: 5 % A, 95 % B; 2.5–5 min: 25 % A, 75 % B; 5–20 min: linear decrease of B from 75 to 30 %, rest A). After each measurement, a washing step was carried out (0–5.5 min: linear increase of C from 75 to 95 %, rest A; 5.5–15 min: 5 % A, 95 % C; 15–20 min: 5 % A, 95 % B). The eluents were A) acetonitrile; B) 25 mM KH_2_PO_4_ (pH 3.5 with 1 M H_3_PO_4_); and C) MiliQ water. Samples were centrifuged prior to analysis and 10 µl undiluted supernatant was injected for HPLC analysis.

### 2.5 Genetic Analysis

#### 2.5.1 Whole Genome and Amplicon Sequencing

For the analysis of whole genome sequences, samples were taken from the bioreactor and frozen to -20°C until the samples were transported to the lab in Vienna under dry ice conditions. Upon arriving in our lab, the samples were stored again at -20°C. For DNA isolation, 2 ml samples were centrifuged for 30 s, with 11,000 x g at room temperature and the pellet was treated with a DNA extraction kit (DNeasy® UltraClean® Microbial Kit, Qiagen, Netherlands) according to the manufacturer’s recommendation. Whole genome sequencing was done by Microsynth AG (Switzerland) using purified genomic DNA samples obtained from biomass samples taken after 2 and 201 days of cultivation. For the Amplicon Deep Sequencing, purified genomic DNA from biomass samples was sent directly to the external laboratory (Microsynth AG, Switzerland). To that end, a Nextera two-step PCR was done using 515f and 806r as forward and reverse primers, respectively (Table 1). The products were sequenced with an Illumina MiSeq. Raw sequencing data were uploaded to the SRA database (accession number PRJNA813737).

**TABLE 1:** Primers used for genetic analyses.

Primer	Sequence (5′−3′)
515f	GTG YCA GCM GCC GCG GTA A
806r	GGA CTA CNV GGG TWT CTA AT
27f	AGA GTT TGA TCC TGG CTC AG
HFX41f	CGA TTT AGC CAT GCT AGT TG
1494r	CTA CGG CTA CCT TGT TAC GA

#### 2.5.2 16s rRNA Sequencing

For sequencing 16S rRNA, 2 ml samples were centrifuged for 10 min with 10,000 rpm at 4°C. the supernatant was discarded, and the pellet was frozen at -20°C until further use. Genomic DNA was isolated from frozen pellet samples using the DNeasy® Kit (UltraClean® Microbial Kit, Qiagen, Netherlands) according to the manufacturer’s recommendation. Subsequently, 16s rDNA was amplified using primers 27f/HFX41f and 1494r (Table 1). Primers were purchased from Integrated DNA Technologies (IDT, Belgium). Sanger sequencing was done by Microsynth Austria GmbH (Austria).

## 3 Results and Discussion

### 3.1 Identification and Characterization of a Halophilic Consortium Found at an Industrial Production Site

This study investigated the effect of scale-up, changing RPB composition and long-term cultivation effects on the process behavior and degradation efficiency of a biological RPB treatment process. To that end, the pilot-scale bioreactor filled with 15 L RPB from the MDA production was inoculated with 1 L of a *H. mediterranei* preculture grown in a lab-scale bioreactor (Mainka et al., 2019). The process started with a batch phase followed by a continuous process phase with cell retention for biomass propagation and to establish a stable growing continuous culture. The experimental phase included the use of weekly changing RPB batches, applying different dilution rates (D) and substrate concentrations for glycerol. In total, the cultivation process was operated for more than 200 days (>4800 h). In addition to process performance, genetic stability was investigated by taking samples for subsequent genome analysis throughout the cultivation.

**FIGURE 2:**
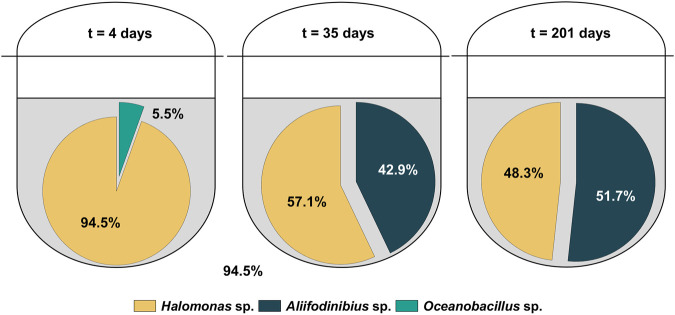
Readout distribution from Amplicon Deep Sequencing of the halophilic microbial community after 4, 35 and 201 days of cultivation.

Specifically, genomic DNA from two biomass samples (days 2 and 201) were sequenced. Surprisingly, the analyzed biomass samples did not show the presence of *H. mediterranei*. Instead, several different bacterial strains mostly from the halophilic bacterial genera of *Halomonas* were identified. This finding was unexpected, as the process performance (organic contaminants were degraded in the measured samples) and growth behavior did not show unusual behavior indicative for a potential contamination. The pre-culture could be excluded as the root cause of the contamination as 16S rRNA analysis showed a pure culture of *H. mediterranei*. To further investigate and verify these results, Amplicon Deep Sequencing was performed, using biomass samples from three different cultivation time points, representing the early (day 4), intermediate (day 35) and final (day 201) stage of the cultivation. To avoid any biased results, no additional cultivation step was performed between the sampling and the DNA analysis. For the Amplicon Deep Sequencing, 16S rRNA (primers 515f and 806r, see Table 1) is only partially amplified. Hence, only genera rather than single species could be identified. Figure 2 shows the results of the Amplicon Deep Sequencing, indicating the percentage of readouts for the identified taxonomic genera. At day 4, the main part of readouts belonged to the bacterial genus *Halomonas* (>94%) while only a minor portion of readouts was identified as either *Oceanobacillus* sp. or *Aliifodinibius* sp. However, the composition of the novel halophilic mixed culture changed over time, as the main percentage of total readouts are allocated to *Halomonas* sp. and *Aliifodinibius* sp. after 35 and 201 days of cultivation. In contrast, the genus of *Oceanobacillus* sp., made up only 0.03 and 0.01% of total readouts at days 35 and 201, respectively. Therefore, it appears that the environment in the bioreactor favors the growth of *Aliifodinibius* and *Halomonas* strains over that of *Oceanobacillus* strains. All the identified genera belong to halophilic (*Halomonas* sp. and *Aliifodinibius* sp.) or halotolerant (*Oceanobacillus* sp.) bacteria (Lu et al., 2001; Mata et al., 2002; Takami et al., 2002; García et al., 2004; Kaye et al., 2004; Wang et al., 2013; Yin et al., 2015; Xia et al., 2016; Cho et al., 2017). To the best of the authors knowledge, this is the first time that a halophilic mixed culture consisting of these three halophilic genera (*Oceanobacillus* sp., *Halomonas* sp. and *Aliifodinibius* sp.) is described for the use in a biological RPB treatment process. The discovery and usage of novel isolated halophilic microorganisms and their exploitation in a biotechnological and industrial context was already successfully demonstrated (Guven et al., 2018; Rezaei Somee et al., 2018; Zare et al., 2019; Hasanzadeh et al., 2020; Villanova et al., 2021). Besides, the usage of a co-culture might be even beneficial for the present bioprocess. Moreover, it was reported for co-cultivations of microorganisms, that productivities and process efficiencies can be improved, compared to monocultures (Angell et al., 2006; Ishika et al., 2017; Anh et al., 2021). Moreover, the potential of halophilic consortia for the bioremediation of saline wastewater contaminated with organic impurities like aromatics was already proven in several cases (Jamal and Pugazhendi, 2018; Al-Shaikh and Jamal, 2020; Jamal and Pugazhendi, 2021; Jin et al., 2021).

To further investigate and characterize the mixed culture found in bioreactor samples, 16S rRNA sequencing experiments were performed, using genomic DNA purified from single colonies. These single colonies were obtained, as a culture sample from the end of the cultivation was grown on agar plates with undefined preculture medium. In total, nine single colonies were re-streaked on a fresh agar plate. From this plate, shake flasks were inoculated, genomic DNA was extracted and used as template for PCR amplification of the 16S rDNA. The results of the 16S rRNA sequencing are summarized in Supplementary File S1 (see Supplementary Table S1).

According to 16S rRNA sequences, all nine single colonies were identified as *Halomonas organivorans* strain G-16.1 which is in line with the results from Amplicon Deep Sequencing where mainly *Halomonas* sp. and *Aliifodinibius* sp. were present in the reactor. Additionally, the identification of strain *H. organivorans* strain G16.1 in a salty environment, contaminated with aromatic substances, is not surprising, as this strain was reported to degrade aromatics, such as phenol (García et al., 2004; de Lourdes Moreno et al., 2011). Additionally, other *Halomonas* strains, such as *H. campisalis* or *H. anticariensis* FP35, are known for their ability to degrade various aromatic compounds, such as catechol or polycyclic aromatic hydrocarbons (PAH) (Alva and Peyton, 2003; Piubeli et al., 2012; Haddadi and Shavandi, 2013; Tena-Garitaonaindia et al., 2019; Govarthanan et al., 2020). Moreover, another *Halomonas* strain, namely *Halomonas* sp. strain MA-C, was previously reported to be able to degrade formate (Oren et al., 1992; Azachi et al., 1995; Heckroth et al., 2018).

The number of readouts for *Aliifodinibius* surpassed that of *Halomonas* sp. at day 201 of the cultivation. Generally, the discovery of *Aliifodinibius* species in the mixed culture and the fact that it become a major part of this culture is surprising as there are only a limited number of publications describe the genus *Aliifodinibius* (Wang et al., 2013; Hahnke et al., 2016; Xia et al., 2016; Cho et al., 2017; Cho and Whang, 2020; Zhao et al., 2020). Members of the genus *Aliifodinibius* include *Aliifodinibius roseus*, *A. sediminis* and *A. halophila* (Wang et al., 2013; Xia et al., 2016). To the best of our knowledge, so far none of the reported strains have been described to use aromatic compounds for growth or the ability to degrade aromatic compounds. The identification of this genus as part of the halophilic mixed culture, however, indicates the ability of *Aliifodinibius* sp. to adapt to the salt concentrations present in the RPB stream and its potential ability to grow on either glycerol or formate. Nevertheless, it is unclear which role this genus has played regarding the degradation of aromatic compounds, or if its presence is beneficial for the use of aromatic compounds by the other genera of *Halomonas* and *Oceanobacillus*.

The reason why the microbial composition in the bioreactor changed between sampling points could be, that a new substrate (glycerol) was introduced into the environment of the bacteria, resulting in a wash-out of cells growing slower with glycerol. Therefore, a halophilic strain could possibly replace all other strains in a bioreactor if its affinity to the growth substrate is the highest for process conditions and the media composition used here.

As *H. organivorans* was identified in the halophilic mixed culture, its ability to grow in the presence of RPB was further investigated. Therefore, shake flask experiments with industrial RPB and glycerol as a co-substrate were performed (results not shown). *H. organivorans* was able to degrade all present organic contaminants (aniline, phenol, MDA, and formate). Compared to the mixed culture, a pure culture of *H. organivorans* grew slower and to a lower maximum optical density. Moreover, during growth of *H. organivorans*, the formation of precipitates could be observed (Supplementary Figure S1A) and were investigated with light microscopy (Supplementary Figure S1B).

**FIGURE 3:**
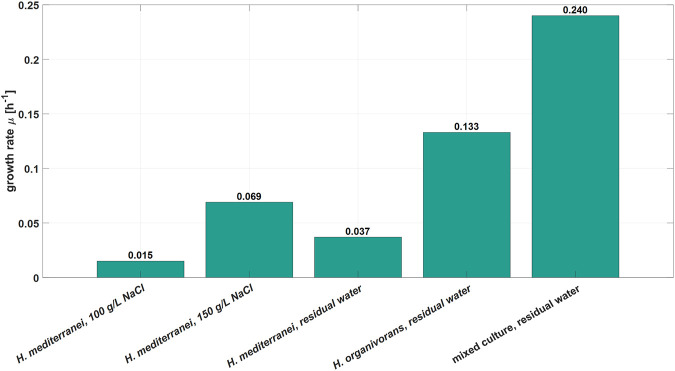
Comparison of specific growth rates between *H. mediterranei*, *H. organivorans*, and the novel mixed culture in the industrial residual process brine. Additionally, *H. mediterranei* was grown in synthetic medium with 100 and 150 g L-1 NaCl.

Furthermore, the growth rates, based on OD_600_ measurements, of the mixed culture and pure cultures of *H. organivorans* and *H. mediterranei* in the RPB were compared. Additionally, *H. mediterranei* was grown in synthetic RPB containing two different NaCl concentrations (100 and 150 g L^−1^). Overall, *H. mediterranei* showed the lowest specific growth rate of the three microbial systems tested, independently of the growth medium (Figure 3). In contrast, the novel mixed culture showed the highest growth rate of 0.24 h^−1^. Consequently, it appears likely that the initial growth conditions together with its general growth advantage favoured the growth of the bacterial mixed culture identified in this study which led to the replacement of *H. mediterranei*. The origin of the contamination could not be clearly identified. However, one plausible explanation might be that the members of the mixed culture found in the reactor grew at different locations of the MDA production site and were introduced to the bioreactor system and its periphery by the operator. As the system periphery could not be heat sterilized, the bacterial contamination was present in the bioreactor at the time of the inoculation and due to a higher level of adaptation to the process conditions, the mixed culture became dominant in the bioreactor.

### 3.2 Process Performance of Halophilic Mixed Culture for the Degradation of Organic Contaminants in an Industrial Residual Process Brine

To evaluate the process performance of the pilot-scale cultivation presented in this study, performance parameters and their acceptance criteria need to be defined. Moreover, the results should be compared with an already established process, treating the same RPB (Mainka et al., 2019). As process variables the dilution rate D, retention rate R and the substrate feeding r_S_ were used. For evaluation of process performance, the efficiency of organic substance degradation was selected. Key process parameters were kept constant during the whole cultivation time: reactor temperature T (= 30 °C), pH (= 7), reactor volume V_R_ (= 16 L) and aeration rate F_air_ (= 0.2 vvm).

As mentioned before, the present study was divided into several process phases. During the first phase (day 1 to 13) the goal was to establish a stable operation of the cultivation setup, including the feed preparation devices. To continue, during a second phase of around 17 days, different dilution rates D (0.1–0.2 h^−1^) and glycerol concentrations (0.5–4 g L^−1^) in the RPB feed were applied to the system. The goal was to maintain a constant biomass level by adjusting the retention rate R accordingly. In the third phase of the study, long-term performance of the process was evaluated by applying in total 23 different RPB batches. In this phase, the dilution rate D was decreased to 0.0625 h^−1^, to apply a weekly change of the RPB feed batch. In this phase, the glycerol concentration was maintained between 1.5 and 2 g L^−1^, and R was adjusted between 0.925 and 0.95 to maintain a biomass concentration constant.

**FIGURE 4:**
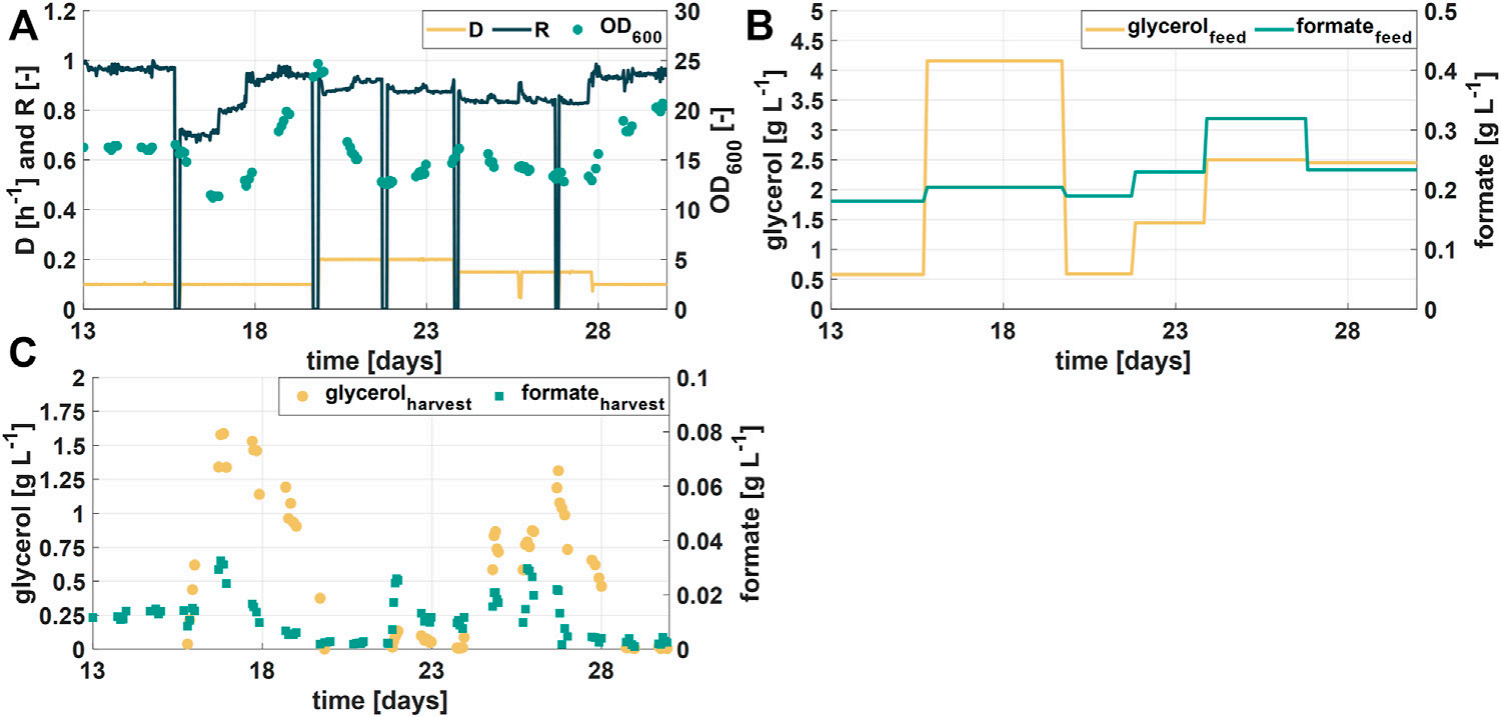
Process performance during the second experimental phase **(A)** Optical density (OD_600_) during the cultivation time of the second phase (17 days). Process parameters (dilution rate D and retention rate R) were changed to maintain a stable biomass concentration **(B)** Feed concentrations of the additional substrate glycerol and formate **(C)** Concentration of glycerol and formate in harvest samples.

Figure 4A shows OD_600_ values, the dilution rate D, and the retention rate R of the second experimental phase (days 13–30) over a course of 17 days of continuous fermentations and Figure 4B shows the variations in the glycerol concentration in the feed. Formate concentrations of six different RPB batches varied between 0.2 and 0.32 g L^−1^ (Figure 4B). Interestingly, during this process phase glycerol accumulation in harvest samples together with decreasing biomass concentrations in the bioreactor was observed when the glycerol concentration in the feed was 4 g L^−1^, operating at D of 0.1 h^−1^ (days three to six, Figure 4C). Simultaneously, the formate degradation decreased, indicated by higher formate concentrations in the harvest. The apparent substrate overfeeding could be linked to the specific growth rate of the culture. Decreasing the specific growth rate by increasing the retention rate R resulted in higher OD_600_ values (days three to six, Figure 4A) and lower glycerol and formate concentrations in the harvest samples (Figure 4C). The results further showed that integrated control of the process variables D, R and r_S_ results in a stable operation of a continuous bioprocess. Moreover, the highest tested dilution rate D (= 0.2 h^−1^) combined with the lowest glycerol feed concentration (= 0.5 g L^−1^) showed the best formate degradation results (>98% of efficiency) while no glycerol was accumulated (days six to eight, Figure 4C). Consequently, further investigations for identification of an optimal glycerol feeding strategy for obtaining high formate degradation would be beneficial for the process efficiency.

**FIGURE 5:**
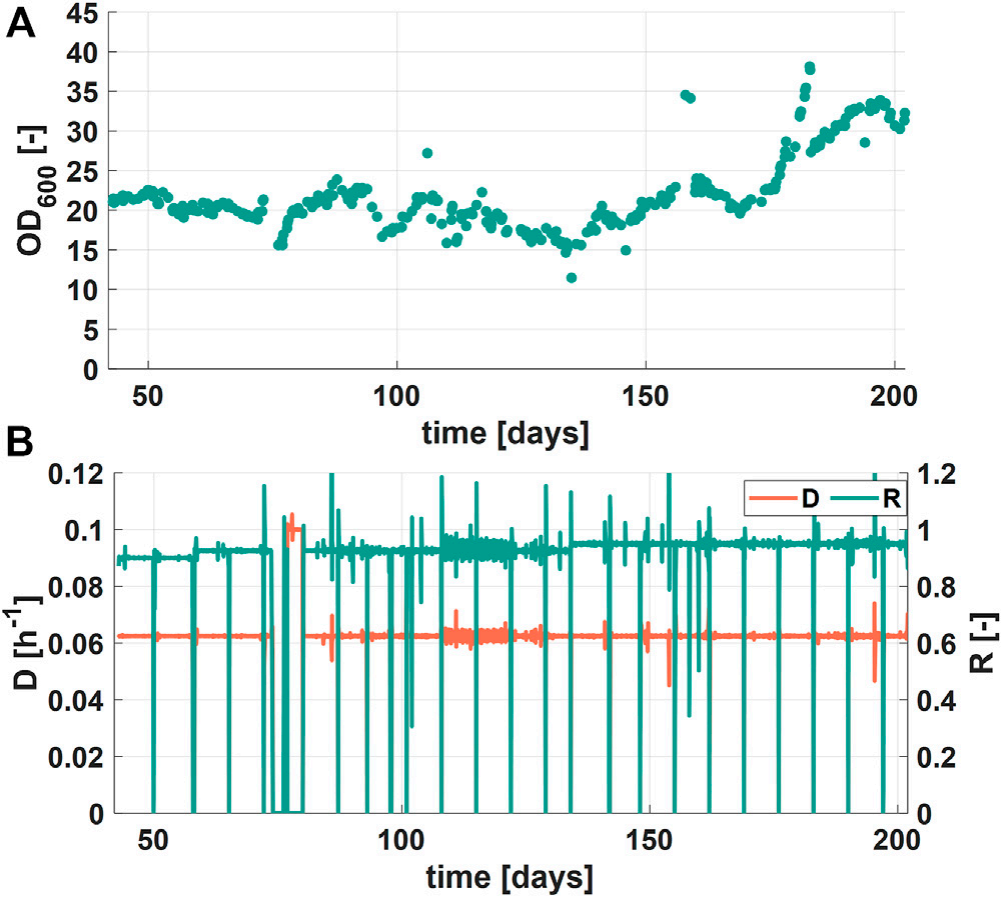
Process performance of long-term experiment phase (days 42–202) **(A)** Optical density over the cultivation time **(B)** Dilution rate D (blue, lower curve) and retention rate R (orange, upper curve) over the cultivation time.

During the third phase (days 42–202), process variables (r_S_, R, D) were chosen in a way that no accumulation of glycerol could be observed. Over the period of these 160 days, the OD_600_ values showed stable values between 15 and 25 (Figure 5A). However, process operation was disturbed due to operational failure of the process control system during two periods (days 73–75 and 182–182). A reason for the increase of OD_600_ to values over 30 during the last 25 days (days 177-202) could not be clearly identified but was probably a result of a failure of the temperature control system (caused by high environmental temperatures). This failure could have either led to higher growth rates or to a change in the morphology of the cells, resulting in potentially larger cells. The dilution rate D, as can be seen in Figure 5B, shows a phase of higher values (0.1 h^−1^). The value was set to 0.1 h^−1^ to consume all the RPB feed left, in order to prepare the new RPB batch according to the time schedule.

As one goal of this study was to investigate the long-term stability of the halophilic bioprocess, particular attention was paid to the influence of the changing contaminant concentrations on process performance. Among the present organic contaminants, phenol and MDA showed stable concentration values between all measured RPB batches (phenol: 3–6 mg L^−1^, MDA: 0.12–0.30 mg L^−1^). In contrast, aniline and formate could vary significantly (aniline: 0.5–15 mg L^−1^, formate: 200–500 mg L^−1^) in their initial feed concentrations.

Nonetheless, HPLC analysis during the long-term experiment showed no detection of residuals of the aromatic compounds aniline, phenol, and MDA in harvest samples. It is assumed that aniline, phenol, and MDA are finally metabolized to CO_2_ through the TCA cycle, as described for halophilic bacteria (Wells and Ragauskas, 2012; Arora, 2015). However, during the metabolism of aromatic substances, intermediates might have occurred, as an unidentified peak was detected in the HPLC chromatograms. In contrast, in previous studies using MDA residual process brine in biological treatment processes, no intermediate accumulation was described (Mahler et al., 2018; Mainka et al., 2019). The peak detected during HPLC analysis with a retention time of 6.8–6.9 min could not yet be identified, thus it is referred to as *unknown substance*. The peak area of the *unknown substance*, however, showed a correlation with the concentration of aniline in the RPB feed (see Supplementary Figure S2). Thus, it is assumed that the *unknown substance* is formed during the degradation of aniline and might be an intermediate of the degradation pathway of aniline.

**FIGURE 6:**
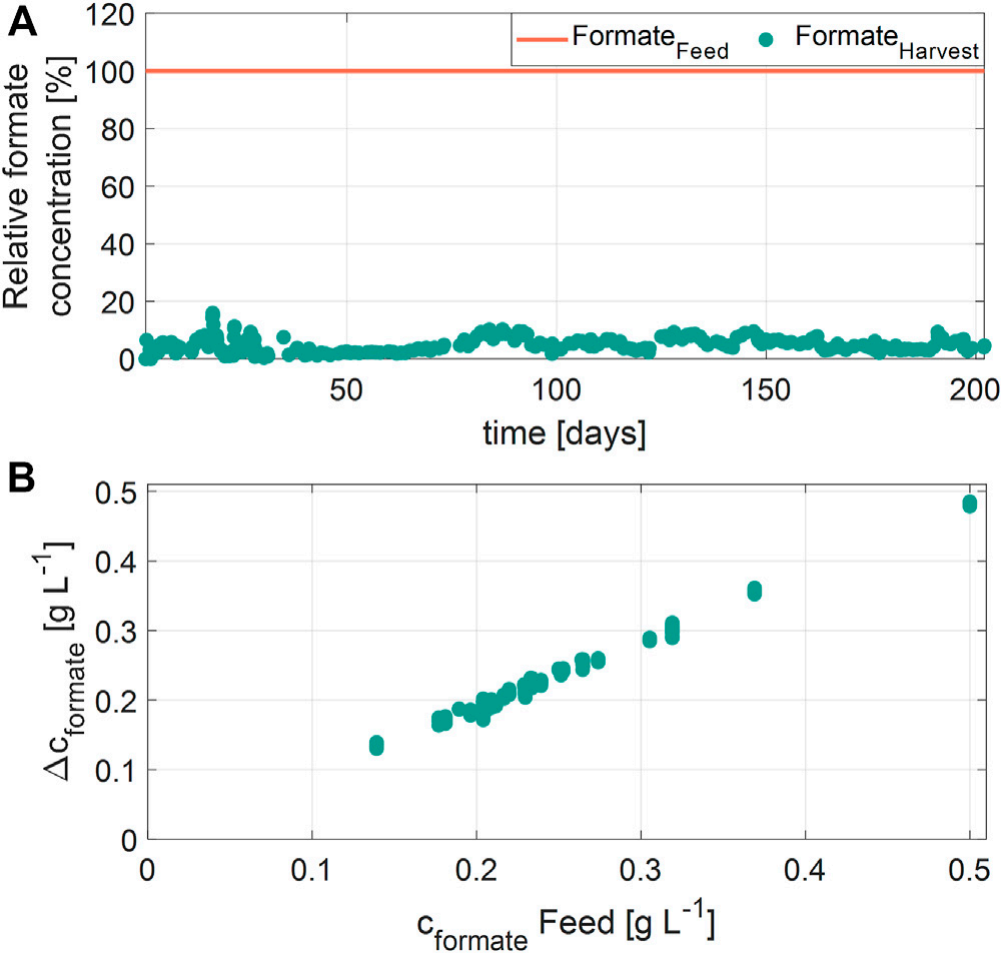
Formate degradation during the cultivation. **(A)** Relative concentrations of formate in the residual process brine feed and harvest samples over time **(B)** Correlation of formate degradation (Δc_formate_) and the formate concentration in the residual process brine (c_formate_ Feed).

Besides, formate was degraded with an efficiency of 90-98%, independently of the concentration in the RPB feed (Figure 6A). However, the absolute amount of formate degraded (Δcformate) showed a linear correlation to the concentration of formate in the feed (Figure 6B). No correlation between the formate degradation and RPB feed concentrations of glycerol, aniline, phenol, or MDA were found. Moreover, the residual concentrations of formate in the harvest were constantly between 10 and 20 mg L^−1^ over the entire cultivation time. It is assumed that formate is metabolized to CO_2_, catalyzed by a formate dehydrogenase (Maia et al., 2017; Yu et al., 2017). Previously, the halophilic bacterium *Halomonas* strain sp. MA-C was shown to degrade formate (Oren et al., 1992; Azachi et al., 1995; Heckroth et al., 2018). Similar to the present study, it was reported, that the total amount of formate degraded by *Halomonas* strain sp. MA-C is dependent on the initial formate concentration, and the NaCl concentration (Heckroth et al., 2018).

In addition to formate oxidation, formate dehydrogenases are able to catalyze the reduction reaction from CO_2_ to formate. The results shown in the present study might indicate that formate is not removed completely because an equilibrium with no further degradation is reached at values of 10–20 mg L^−1^ formate in the bioreactor (Maia et al., 2017; Yu et al., 2017). Furthermore, previous reports for *Halomonas* strain sp. MA-C showed that less than 5% of C^14^-labeled formate was incorporated into cells. Therefore, it appears likely that formate plays only a minor role in biomass formation (Oren et al., 1992; Azachi et al., 1995).

### 3.3 Comparison of Scale-Up Process to Existing Bioprocesses for MDA Residual Process Brine Treatment

In two previously published studies, RPB from MDA-production was biologically treated using the extremely halophilic archaeon *H. mediterranei* in a 1L lab-scale stirred tank reactor and a 16L bubble column reactor (Mahler et al., 2018; Mainka et al., 2019). There, the goal was also to reduce the organic load in the RPB for later reuse in base chemical production. The previous lab-scale study showed that 100% reduction of the present organic contaminants was possible for a cultivation time of over 54 days (Mainka et al., 2019). However, only one RPB batch was used, as the study focused on the influence of different process variables like D, R, and the glycerol feed concentration. In contrast to previous results, the present study investigated the influence of batch-to-batch variability of RPB on process performance. Moreover, higher values for the dilution rate D and the retention rate R, and lower glycerol feed concentrations were tested, key parameters to optimize overall process economy. High values for D and R increase the process productivity by increasing the capacity of the RPB reduces costs as less unused bleed stream is generated. Furthermore, decreased glycerol consumption improves overall process economics.

The previously published pilot-scale study showed the technical feasibility of using a bubble column reactor for the growth of *H. mediterranei* in salty RPB. However, the cultivation time of 35 h was short compared to the cultivation time in the present study. The process in the present study, therefore, combined and extended the previously gained knowledge, by scaling-up the previous process to a higher volume of 16L and an extended cultivation time of over 210 days. Moreover, for the first time it was achieved to directly integrate the process in the RPB production on the MDA production site. Table 2 compares the process parameters and variables of this study with the previously published lab-scale process.

**TABLE 2:** Comparison of scale-up performance.

Parameter	Lab-scale	Pilot-scale
References	(Mainka et al., 2019)	(this study)
Reactor volume [L]	1	16
Microorganisms/strain	*H. mediterranei*	Halophilic mixed culture
No. of RPB batches	1	32
Temperature [°C]	37	30
pH	7.00	7.00
Dilution rate D [h−1]	0.1	0.0625-0.2
Retention rate R [-]	0.74-0.87	0.7-0.95
Glycerol concentration [g L−1]	2-10	0.5-2.5
Glycerol feeding rate [g L−1 h−1]	0.2-1.0	0.05-0.4
Formate removal [%]	100%	90–98%
Aromatics removal [%]	100%	100%

Different from the previous study, in this work the process temperature could be lowered to 30°C (from 37°C). Lower process temperatures are not only less energy consuming but also water evaporation during the process can be reduced. Furthermore, the present study showed that compared to the lab-scale study, simultaneous application of low glycerol feeding with high dilution and retention rates resulted in similar degradation rates for organic impurities. As a result, lower consumption of the additional carbon glycerol was observed, which could help to lower operational costs. Additionally, a reduced use of the additional carbon source necessitates a higher retention rate R to maintain the same biomass level. As already mentioned before, high R values in turn result in a reduced bleed stream volume. Therefore, less waste stream is generated as the bleed stream is currently not further used in the process setup. However, a potential future use of the bleed stream, e.g., by burning the biomass for heat generation, could further increase the process sustainability.

In addition to the bioreactor size and the energy input to the system, the bioprocess presented in this study differs mainly from previous studies in the microbial system used for degradation of organic pollutants. During this study, a halophilic community was identified which proved to be able to degrade all organic impurities in the RPB with high efficiency. Compared to *H. mediterranei*, the mixed culture has a higher growth rate when grown in the same RPB stream. The mixed culture found in this study consisted of halophilic bacteria which might be more flexible towards changing NaCl concentrations compared to *H. mediterranei*, especially at lower NaCl levels. In detail, salt tolerance in bacteria is based on the production of compatible solutes which could potentially provide an advantage to bacteria over archaea (relying on the “salting-in” strategy) to deal with changing salt concentrations (Oren, 2002; Mainka et al., 2021). Nevertheless, there is most likely an optimal range of the NaCl concentration, where the halophilic bacterial mixed culture most efficiently degrades formate and aromatic compounds.

## 4 Conclusion and Outlook

For the first time, a pilot-plant bubble column bioreactor system was successfully implemented at an industrial MDA production site and operated continuously for more than 210 days. Process parameters were compared to a small-scale process using RPB from the same production site. The results achieved with in the pilot-scale bioreactor showed degradation efficiencies as high as in the lab-scale bioreactor process. In this study, the bioreactor initially inoculated with *H. mediterranei*, was contaminated with a halophilic mixed culture, consisting of at least three different bacterial genera (*Halomonas* sp., *Aliifodinibius* sp. and *Oceanobacillus* sp.). The bacterial mixed culture could replace the original *H. mediterranei* culture possibly due to a better adaptation to the RPB stream. The identified microbial community was highly adapted to the environment of the industrial production site and showed high degradation efficiencies for the contaminants present in the RPB. Compared to *H. mediterranei*, the halophilic community also showed higher growth rates when grown in RPB. Nevertheless, additional investigations of the optimal salt concentration ranges for the degradation of organic contaminants in RPB treatment processes are required for an improved process understanding. Concentrations of aromatics in the RPB feed were changing significantly between the batches. The results showed that aniline concentrations in the RPB feed are positively correlated to the accumulation of a potential, yet unknown intermediate. So far it is not known if this intermediate is affecting the process efficiency of a chlor-alkali electrolysis process. Nevertheless, future investigations should be done to identify the substance and its effect on the efficiency of the membrane-cell chlor-alkali electrolysis process. Formate degradation showed a dependency on the formate concentration in the RPB feed. However, residual concentrations of formate were always between 10 and 20 mg L^−1^. Moreover, if the additional substrate glycerol was overfed and thus accumulating in the bioreactor, also the degradation efficiency for formate was decreasing. The results of the present study further indicated a correlation between the glycerol feeding and the formate degradation, as at the lowest glycerol concentration combined with the highest dilution rate resulted in the lowest residual formate concentration. To increase process flexibility, the RPB feed could be decoupled from the co-substrate feeding by adding an additional glycerol feed to the system. Consequently, biomass concentration could be adapted based on fluctuating aromatic concentrations independently from the retention rate R solely by changes in the glycerol feed. Furthermore, a potential correlation of the glycerol feeding, and the formate degradation should be investigated to minimize the use of glycerol and to optimize the formate degradation efficiency. In conclusion, the present study underlines the potential of an alternative and sustainable bioprocess for treating residual process brine, and once more emphasizes the possibilities natural microbial diversity offers for exploitation in an industrial context.

## Data Availability

The datasets presented in this study can be found in online repositories. The names of the repository/repositories and accession number(s) can be found below: https://www.ncbi.nlm.nih.gov/, PRJNA813737.
