# Corrigendum: Reducing Organic Load From Industrial MDA Residual Process Brine With a Novel Halophilic Mixed Culture: Scale-Up and Long-Term Piloting of an Integrated Bioprocess

**DOI:** 10.3389/fbioe.2022.926194

**Published:** 2022-05-13

**Authors:** Thomas Mainka, Christoph Herwig, Stefan Pflügl

**Affiliations:** ^1^ Institute for Chemical, Environmental and Bioscience Engineering, TU Wien, Vienna, Austria; ^2^ Competence Center CHASE GmbH, Linz, Austria

**Keywords:** bioprocess scale-up, halophilic microbial community, industrial residual process brine treatment, aromatic degradation, long-term bioprocessing

In the original article, “we neglected to acknowledge TU Wien Bibliothek for financial support through its Open Access Funding Programme.”

The authors apologize for this error and state that this does not change the scientific conclusions of the article in any way. The original article has been updated.

