# Supplementary material for: Reducing Organic Load From Industrial Residual Process Brine With a Novel Halophilic Mixed Culture: Scale-Up and Long-Term Piloting of an Integrated Bioprocess
Source: Front Bioeng Biotechnol. 2022 Apr 19;10:896576. doi: 10.3389/fbioe.2022.896576 (PMC9062027; doi:10.3389/fbioe.2022.896576)
Supplement: Supplementary file 1 [file DataSheet1.docx]

Supplementary Material

# Supplementary Data

Supplementary Material should be uploaded separately on submission. Please include any supplementary data, figures and/or tables. All supplementary files are deposited to FigShare for permanent storage and receive a DOI.

Supplementary material is not typeset so please ensure that all information is clearly presented, the appropriate caption is included in the file and not in the manuscript, and that the style conforms to the rest of the article. To avoid discrepancies between the published article and the supplementary material, please do not add the title, author list, affiliations or correspondence in the supplementary files.

# Supplementary Figures and Tables

## Supplementary Figures

**Supplementary Figure 1.** Precipitates of *Halomonas organivorans* growing in shake flaks. A) Image of the shake flasks with visible agglomerates of H. organivorans. B) Image of *H. organivorans* agglomerates under the microscope (1000x).

Supplementary Figure 2. HPLC chromatogram peak are of an unidentified, potential intermediate during aniline degradation.

## Supplementary Tables

Supplementary Table 1. Results of 16S rRNA sequencing experiments.

| **Contig #** | **rRNA cover** | **Query cover [%]** | **Identity [%]** | **Strain** |
| --- | --- | --- | --- | --- |
| 1 | 49-1462 | 100 | 99,86 | *Halomonas organivorans* strain G-16.1 |
| 2 | 49-1462 | 100 | 99,86 | *Halomonas organivorans* strain G-16.1 |
| 3 | 49-1462 | 100 | 99,86 | *Halomonas organivorans* strain G-16.1 |
| 4 | 49-1462 | 100 | 99,86 | *Halomonas organivorans* strain G-16.1 |
| 5 | 49-1462 | 100 | 99,86 | *Halomonas organivorans* strain G-16.1 |
| 6 | 49-1462 | 100 | 99,86 | *Halomonas organivorans* strain G-16.1 |
| 7 | 49-1462 | 100 | 99,86 | *Halomonas organivorans* strain G-16.1 |
| 8 | 49-1462 | 100 | 99,86 | *Halomonas organivorans* strain G-16.1 |
| 9 | 49-1462 | 100 | 99,86 | *Halomonas organivorans* strain G-16.1 |
